# The Feasibility, Safety and Outcome of Very High-Power Short Duration Radiofrequency Ablation in Pulmonary Vein Isolation: A Real-World Observation Study

**DOI:** 10.31083/j.rcm2507250

**Published:** 2024-07-05

**Authors:** Akash Mavilakandy, Ivelin Koev, Bharat Sidhu, Ahmed Kotb, Ibrahim Antoun, Sharon H Man, Zakariyya Vali, Vivetha Pooranachandran, Joseph Barker, Gaurav Panchal, Xin Li, G. André Ng

**Affiliations:** ^1^Department of Cardiology, University Hospitals of Leicester NHS Trust, LE1 5WW Leicester, UK; ^2^Department of Cardiovascular Sciences, University of Leicester, LE1 7RH Leicester, UK; ^3^National Institute for Health Research Leicester Biomedical Research Centre, LE3 9QP Leicester, UK; ^4^School of Engineering, University of Leicester, LE1 7RH Leicester, UK

**Keywords:** atrial fibrillation, pulmonary vein isolation, radiofrequency ablation, very-high-power-short duration ablation, COVID-19

## Abstract

**Background:**

Pulmonary vein isolation (PVI) ablation is the established gold standard therapy for patients with symptomatic drug refractory atrial fibrillation (AF). Advancements in radiofrequency (RF) ablation, have led to the development of the novel contact force-sensing temperature-controlled very high-power short-duration (vHPSD) RF ablation. This setting delivers 90 W for up to 4 seconds with a constant irrigation flow rate of 8 mL/min. The aim of this study was to compare procedural outcomes and safety with conventional radiofrequency ablation.

**Methods:**

An observational study was conducted with patients who underwent first time PVI ablation between August 2020 and January 2022. The cohort was divided into: (1) vHPSD ablation; (2) High-power short duration (HPSD) ablation; (3) THERMOCOOL SMARTTOUCH™ SF (STSF). The vHPSD ablation group was prospectively recruited while the HPSD and STSF group were retrospectively collected. Primary outcomes were procedural success, PVI duration, ablation duration and incidence of perioperative adverse events. Secondary outcomes were intraprocedural morphine and midazolam requirement.

**Results:**

A total of 175 patients were included in the study with 100, 30 and 45 patients in the vHPSD, HPSD and STSF group, respectively. PVI was successfully attained in all vHPSD patients. vHPSD demonstrated significantly reduced time required for PVI and total energy application in comparison to the HPSD and STSF groups (67.7 ± 29.7 vs. 92.9 ± 25.7 vs. 93.6 ± 29.1 min, *p* < 0.0001; 9.87 ± 4.16 vs. 33.9 ± 7.49 vs. 36.0 ± 10.5 min, *p* < 0.0001, respectively). Intravenous morphine and midazolam requirement was lower in the vHPSD group compared to the HPSD and STSF groups (10.2 ± 3.43 vs. 16.1 ± 4.58 vs. 15.3 ± 3.94 mg, *p* < 0.0001; 4.04 ± 3.24 vs. 8.63 ± 5.22 vs. 8.58 ± 4.72 mg, *p* < 0.0001). One cardiac tamponade was observed in both the vHPSD and HPSD groups while the STSF group exhibited an embolic stoke and two pericardial effusions that did not require drainage.

**Conclusions:**

In this study, vHPSD demonstrated a comparable safety profile to the other treatment arms. Procedural duration and energy application time was substantially reduced along with sedation requirement notwithstanding the limitations of observational study design, these preliminary findings are promising with respect to periprocedural outcomes and safety of vHPSD however longitudinal outcomes will be essential to assessing the overall efficacy of this novel technology.

## 1. Introduction

Atrial fibrillation (AF) is the most common arrhythmia with a worldwide prevalence of approximately 1–2% with serious health complications such as stroke and heart failure [1]. Advancements in the understanding of the underlying pathophysiology have led to the development of various treatment strategies broadly divided into pharmacological and procedural interventions. Catheter ablation forms the foundation for an invasive approach with pulmonary vein isolation (PVI) as the only proven evidence-based invasive procedure for paroxysmal AF [1]. Substrate modification in persistent AF remains an area of ongoing research. PVI is largely performed by radiofrequency ablation (RFA) or cryo-balloon ablation although recently pulsed field ablation (PFA) has emerged as a new energy source for PVI by targeted electroporation of cardiomyocytes [2, 3]. RFA is usually done in conjunction with a 3D electro-anatomical mapping system and employs a point-by-point lesion strategy in accordance with the navigation data from the 3D mapping system. Radiofrequency (RF) catheter technology has undergone continual development over several years with an increased focus on contact force technology to optimise safety and efficiency [4]. The duration of energy application required for individual lesions, however, has led to long procedural times. Thus, a very high power-short duration (vHPSD) strategy for RFA was devised [5]. The University Hospitals of Leicester was one of the first tertiary centres in the UK to introduce the application of QDOT MICRO™ Catheter in QMODE+™ ablation mode for PVI procedures. This technology utilises the principles of vHPSD and operates with 90 W for a maximum duration of 4 seconds for each lesion.

We conducted a real-world observational study comparing procedural time, sedation requirement and procedural outcomes between QDOT MICRO™ Catheter with QMODE+™ (vHPSD) (Biosense Webster, Inc., Irvine, CA, USA), QDOT MICRO™ Catheter with QMODE (HPSD) (Biosense Webster, Inc., Irvine, CA, USA) and THERMOCOOL SMARTTOUCH® SF Catheter with standard RF ablation (STSF) (Biosense Webster, Inc., Irvine, CA, USA).

## 2. Materials and Methods

### 2.1 Study Design

A local registry of patients who had PVI with the use of vHPSD was prospectively observed and analysed. The high-power short duration (HPSD) and STSF groups were retrospectively collated following compatibility with inclusion criteria. Patients over the age of 18 with first time PVI ablation for symptomatic atrial fibrillation were included. Subjects with any previous catheter or surgical ablation were excluded.

This study was registered as a quality improvement project into the current RF technologies utilised for PVI procedures at the University Hospitals of Leicester.

### 2.2 Ablation Procedure

Procedures were either carried out under general anaesthetic (GA) or sedation with local anaesthesia. Patients listed for sedation and local anaesthesia procedures were given intravenous (IV) diazepam 5 mg, morphine 5 mg, metoclopramide 10 mg and 1 g Paracetamol at the start of the procedure. Further bolus doses of IV Midazolam 1–2 mg and morphine were administered as required during the procedure. Venous access was gained via the right femoral vein under ultrasound guidance. All patients underwent 3D electro-anatomical mapping using the CARTO 3 system (Biosense Webster, Inc., Irvine, CA, USA) with either a Lasso or PentaRay catheter (Biosense Webster, Inc., Irvine, CA, USA) following standard trans-septal puncture.

The general ablation strategy for PVI was wide area circumferential ablation (WACA) with substrate ablation conducted in the posterior wall +/– roof +/– anterior wall when indicated.

An open-irrigated tip catheter (QDOT MICRO, Biosense Webster, Inc., Irvine, CA, USA) was utilised. Visualizable steerable sheaths were used by certain operators when required however were not routinely used. The QDOT MICRO ablation catheter incorporates six thermocouples situated in the catheter tip, facilitated for accurate local temperature measurement. The positioning of the electrodes is optimised to monitor the temperature at both perpendicular and parallel catheter orientations which provides the basis for a susceptible feedback system of catheter-tissue interface temperature and thus, catheter stability during energy application [6, 7]. During the RF application, the vHPSD algorithm continually modulates the power based on the greatest surface temperature detected by the thermocouples. The primary setting utilised for the QMODE+ group was 90 W, 4 s; irrigation at 8 mL/min; recommended contact force setting of 5–25 grams. The temperature setting followed a cut-off of 65 degrees Celsius. This approach promotes resistive heating and reduces conductive heating [5, 8, 9].

The QDOT MICRO Ablation catheter was also utilised for conventional ablation in the HPSD patient cohort. Within this mode, the system adjusts the irrigation flow rate and power based on the recorded temperature to stabilize the catheter tip temperature.

The Thermocool Smart-touch SF (Biosense Webster, Inc., Irvine, CA, USA) open-irrigated tip catheter was used for conventional ablation in the STSF patient cohort. Ablation was conducted in the power-control mode with energy application limited to 40 W at the anterior segments and guided by the ablation index (AI) [10].

An activated clotting time (ACT) was maintained between 300–400 ms and was checked regularly (in 15–30 min interval) as per ACT once access to the left atrium was achieved. Operators aimed for an overlap of anterior wall lesions and thus intralesional distance was ≤4 mm, while posterior wall lesions was <6 mm. PVI was confirmed with entrance and exit block using pacing manoeuvres and/or adenosine challenge to confirm absence of pulmonary vein (PV) reconnection. If PV conduction was still apparent, additional lesions were performed where indicated with either the vHPSD or standard power setting (40–50 W in QMODE) respectively if the gap was on the anterior (90 W) or posterior (50 W) portion of the left atrium. The durability of PVI lesions was observed with a wait period of 30 minutes, to ensure there was no further acute reconnection. Cavotricuspid isthmus (CTI) ablation was performed with standard RF ablation technique when typical atrial flutter was documented previously or observed during the procedure. Substrate lesions were performed at the discretion of the operator for persistent AF.

### 2.3 Study Outcomes

The primary outcomes were focused on intraoperative variables corresponding to the following: PVI ablation duration (time between from the first lesion of the WACA to confirmation of PV isolation of the last ablation lesion), duration of energy application for PVI, short-term efficacy of procedure and incidence of procedural adverse events (PAE). The short-term efficacy of the procedure was determined if PVI was confirmed following pacing manoeuvres, adenosine or isoproterenol challenge. The procedural metrics were collected via the CARTO 3 system (Biosense Webster, Inc., Irvine, CA, USA) and LABSYSTEM™ PRO EP Recording system (Boston Scientific, Inc., Marlborough, MA, USA).

The outcome corroborating to safety was derived from the incidence of adverse events which included cardiac tamponade or perforation, significant vascular access complication, stroke, transient ischaemic attack and periprocedural mortality. As per post-procedural management, patients were monitored as inpatients overnight and received a focussed transthoracic echocardiogram to exclude any pericardial effusion immediately after the procedure and prior to hospital discharge.

Secondary outcomes were sedation requirement and fluoroscopy duration. Patient symptoms particularly corresponding to pain and discomfort were routinely reviewed by the procedural team during the intraoperative period. The procedural dosage of IV morphine and IV midazolam was extracted from the procedural drug chart and checked with the catheter laboratory control drug record.

Antiarrhythmic pharmacological management subsequent to the procedure was at the discretion of the operator.

### 2.4 Statistical Analysis

Categorical variables were expressed as frequency and percentage. Median and interquartile range (25th–75th percentiles) was used to describe non-parametric continuous data. Mean and standard deviation was used to describe parametric continuous data. The Shapiro-Wilk test was used to ascertain for normality.

Comparisons between unpaired groups of parametric data was conducted with the Student’s *t*-test while non-parametric data unpaired comparisons were conducted with the Mann-Whitney U test. Categorical variables were compared with the use of χ^2^ test. The one-way analysis of variance (one-way ANOVA) with Bonferroni test for pairwise comparison was used for parametric comparison of three or more groups. The Kruskal-Wallis test with Dunn’s multiple’s comparison test was used for non-parametric comparison of three or more groups. A *p* < 0.05 was considered statistically significant.

Statistical analysis was conducted with the use of GraphPad Prism 8 (GraphPad Software, Inc., San Diego, CA, USA).

## 3. Results

### 3.1 Patient Demographics and Characteristics 

Between August 2020 and January 2022, a total of 100 patients underwent procedures prospectively in the vHPSD group according to the study criteria. Thirty and forty-five patients were retrospectively included in the HPSD and STSF groups, respectively.

Baseline characteristics and demographics are summarised in Table 1. The median age (interquartile range, IQR) of the vHPSD, HPSD and STSF groups were 62.5 (56–69), 61 (53.5–68) and 59 (53–65) with the prevalence of the male gender at approximately 2/3 (71%, 70% and 73%). The prevalence of paroxysmal AF in the vHPSD, HPSD and STSF groups were 67%, 53%, and 58%, respectively. Additionally, the prevalence of persistent AF in the vHPSD, HPSD, and STSF groups were 33%, 47%, and 42%. The median CHA₂DS₂-VASc (IQR) of the vHPSD, HPSD and STSF groups were 1 (1–2), 2 (0–2.25) and 1 (0–2). The mean (confidence interval, CI) left atrial volume index (LAVI) across the vHPSD, HPSD and STSF groups were in the mild category at 29 ± 17.9 ${\mathrm{mL/m}}^{2}$, 30.6 ± 13.1 ${\mathrm{mL/m}}^{2}$ and 32.8 ± 13.6 ${\mathrm{mL/m}}^{2}$, respectively.

**Table 1.** **Patient demographics and characteristics**.

Patient demographics		vHPSD (n = 100)	HPSD (n = 30)	STSF (n = 45)	*p* value
Gender (%)					
	Male	71	70	73	0.942
Age/years		62.5 (56–69)	59 (53.5–65)	61 (53.75–68)	0.488
BMI (${\mathrm{kg/m}}^{2}$)		29.7 ± 0.457	29.8 ± 4.88	31.2 ± 4.82	0.204
CHA₂DS₂-VASc		1 (1–2)	2 (0–2.25)	1 (0–2)	0.669
LAVI (${\mathrm{mL/m}}^{2}$)		29.0 ± 1.79	30.6 ± 13.1	32.8 ± 13.6	0.487
Paroxysmal AF (%)		67	53	58	0.307
Persistent AF (%)		33	47	42	
Medication (%)					
	No current pharmacological therapy (%)	2	0	2.22	0.726
Monotherapy (%)					
	Total	84	60	80	0.0181
	BB	33	20	33.3	0.368
	Calcium channel blocker	2	3.33	0	0.522
	Amiodarone	9	13.3	4.44	0.393
	Flecainide	6	6.67	2.22	0.583
	Sotalol	34	16.7	40	0.0964
Dual therapy (%)					
	Total	14	40	17.8	0.00650
	BB + Flecainide	11	16.7	11.1	0.394
	BB + Amiodarone	3	16.7	6.67	0.0240
	Sotalol	0	6.67	0	0.00750

LAVI, left atrial volume index; BB, beta-blocker; vHPSD, very high power-short duration; HPSD, high-power short duration; STSF, THERMOCOOL SMARTTOUCH™ SF; AF, atrial fibrillation; BMI, body mass index.

### 3.2 Procedural Metrics

**Fig. 1.**
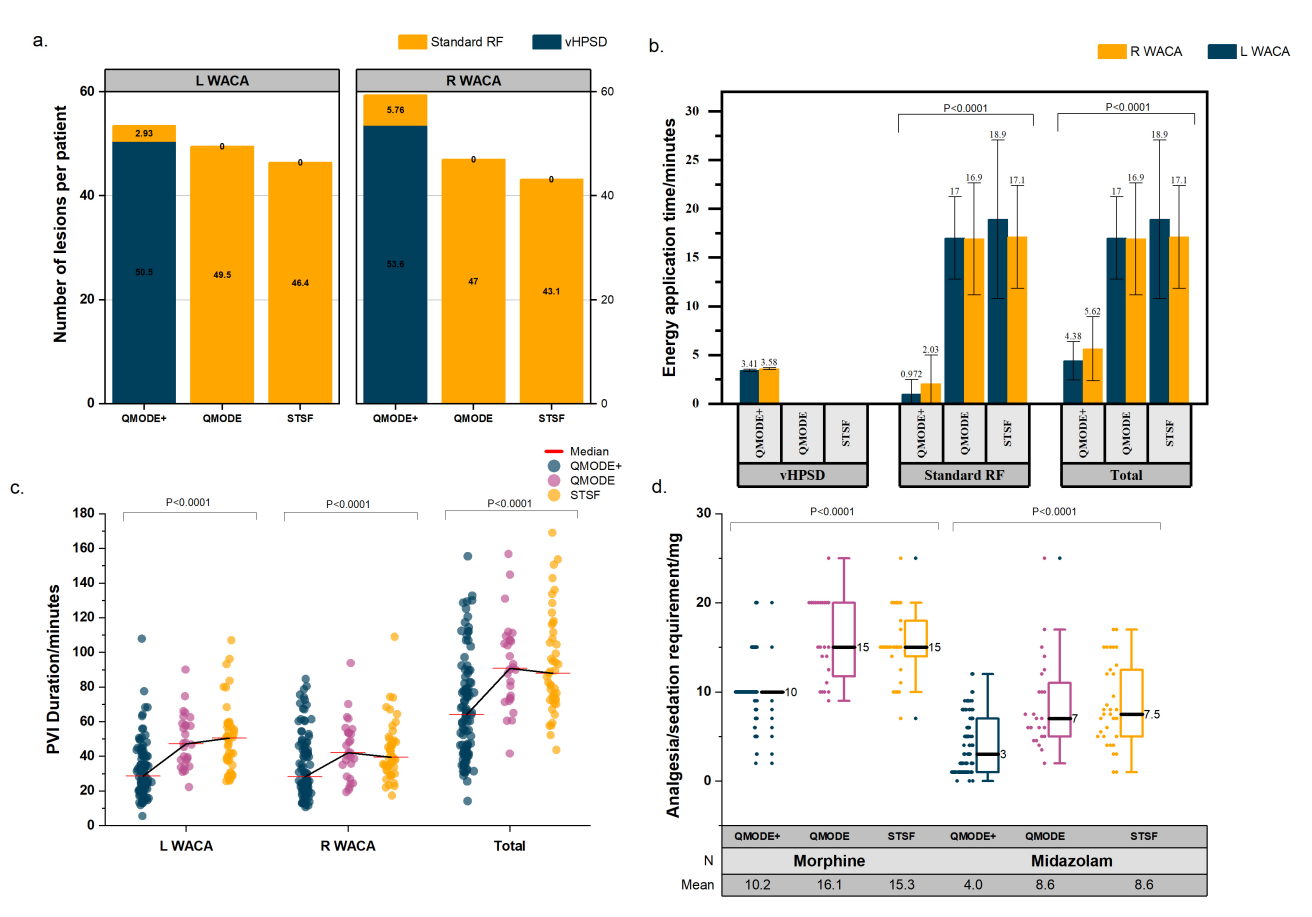
**Procedural outcomes.** (a) Number of lesions per patient. (b) Energy application time. (c) Total PVI duration. (d) Analgesia and sedation requirement. RF, radiofrequency ablation; vHPSD, very high power-short duration; WACA, wide area circumferential ablation; STSF, THERMOCOOL SMARTTOUCH™ SF; PVI, pulmonary vein isolation; L, left; R, right.

Of the patients in the vHPSD, HPSD and STSF group, substrate ablation was performed in addition to PVI in 40 (40%), 17 (56.7%) and 20 (44.4%) patients, respectively. The median number (IQR) of RF energy applications required for PVI alone in the vHPSD group, vHPSD and STSF group was 111 (96.75–129.3), 93 (80.25–112.5) and 85 (74.5–99.5) (*p* < 0.0001) (Table 2) (Fig. 1). Of the vHPSD group, the median (IQR) number of vHPSD and conventional RF lesions were 101 (90.5–119) and 3 (0–14). The mean total RF ablation time for the vHPSD, HPSD and STSF groups were 9.87 ± 4.16 min, 33.9 ± 7.49 min and 36.0 ± 10.5 min (*p* < 0.0001), respectively (Fig. 1). The mean total time required for PVI in the vHPSD, HPSD and STSF groups was 67.7 ± 29.7 min, 92.9 ± 25.7 min and 93.6 ± 29.1 min (*p* < 0.0001). The mean fluoroscopy time corresponding to the vHPSD, HPSD and STSF group was 12.9 ± 8.00 min, 14.1 ± 12.8 min and 16.1 ± 10.1 min (*p* = 0.1981).

**Table 2.** **Intraprocedural metrics**.

Procedural metrics	vHPSD (n = 100)		HPSD (n = 30)		STSF (n = 45)		*p* value
Number of lesions							
PVI							
vHPSD	101 (90.5–119)		N/A		N/A		N/A
Conventional RF power setting	3 (0–14)		93 (80.25–112.5)		85 (74.5–99.5)		<0.0001
Total number of lesions for PVI	111 (96.75–129.3)		93 (80.25–112.5)		85 (74.5–99.5)		<0.0001
Total number of lesions in procedure	128 (101–160.5)		111.5 (87.5–140.5)		103 (88–130)		0.0018
WACA metrics	L WACA	R WACA	L WACA	R WACA	L WACA	R WACA	
Number of vHPSD lesions	49 (40–58.5)	51.5 (41–66)	N/A	N/A	N/A	N/A	N/A
Number of conventional RF lesions	0 (0–4.5)	0 (0–9.5)	45.5 (41.25–58.75)	49 (36.25–57)	43 (35.5–53)	40 (36–50.5)	<0.0001
Total number of lesions	52 (42–64)	59 (44–70)	45.5 (41.25–58.75)	49 (36.25–57)	43 (35.5–53)	40 (36–50.5)	<0.0001
Duration of vHPSD energy application/min	3.41 ± 0.104	3.58 ± 0.0985	N/A	N/A	N/A	N/A	N/A
Duration of conventional RF energy application/min	0.972 ± 1.51	2.03 ± 2.97	17 ± 4.23	16.9 ± 5.74	18.9 ± 8.15	17.1 ± 5.28	<0.0001
Total energy application time/min	4.38 ± 1.97	5.62 ± 3.28	17 ± 4.23	16.9 ± 5.74	18.9 ± 8.15	17.1 ± 5.28	<0.0001
Isolation duration/min	32.4 ± 16.0	34.9 ± 18.9	48.7 ± 15.5	44.1 ± 16.9	50.4 ± 19.7	43.3 ± 17.1	<0.0001
Total energy application time in PVI/min	9.87 ± 4.16		33.9 ± 7.49		36 ± 10.5		<0.0001
Total time required for PVI/min	67.7 ± 29.7		92.9 ± 25.7		93.6 ± 29.1		<0.0001
Fluoroscopy duration/min	12.9 ± 8.00		14.1 ± 12.8		16.1 ± 10.1		0.162

RF, radiofrequency; PVI, pulmonary vein isolation; WACA, wide area circumferential ablation; vHPSD, very high power-short duration; HPSD, high-power short duration; STSF, THERMOCOOL SMARTTOUCH™ SF; N/A, not available/applicable; L, left; R, right.

### 3.3 Sedation Requirement

Within the vHPSD, HPSD and STSF groups, 14, 8 and 2 patients received general anaesthesia. The mean intra-procedural administered dose of intravenous morphine in the vHPSD, HPSD and STSF groups were 10.2 ± 3.43 mg, 16.1 ± 4.58 mg and 15.3 ± 3.94 mg (*p* < 0.0001) in patients who had PVI under conscious sedation (Table 3) (Fig. 1). The mean intra-procedural administered dose of intravenous midazolam in the vHPSD, HPSD and STSF groups were 4.04 ± 3.24 mg, 8.63 ± 5.22 mg and 8.56 ± 4.72 mg (*p* < 0.0001).

**Table 3.** **Procedure outcomes and analgesia/sedation requirement**.

Patient demographics	vHPSD (n = 100)	HPSD (n = 30)	STSF (n = 45)	*p* value
PVI success/%	100	100	100	0.508
Pulmonary vein reconnection/%	45	30	35.6	0.134
Total morphine intraprocedural usage/mg	10.2 ± 3.43	16.1 ± 4.58	15.3 ± 3.94	<0.0001
Total midazolam intraprocedural usage/mg	4.04 ± 3.24	8.63 ± 5.22	8.56 ± 4.72	<0.0001
Periprocedural adverse event (%)	3 (3)	1 (3.33)	4 (8.88)	0.273

vHPSD, very high power-short duration; HPSD, high-power short duration; STSF, THERMOCOOL SMARTTOUCH™ SF; PVI, pulmonary vein isolation.

### 3.4 PVI Efficacy

PVI was successfully achieved in all patients in the study. Fifty-six patients (56%) in the vHPSD group required supplementary conventional RF lesions to attain PVI. First-pass PVI of all PVs was attained in 55 (55%), 21 (70%) and 29 (64%) patients in the vHPSD, HPSD and STSF group, respectively (*p* = 0.134). The vHPSD, HPSD and STSF groups demonstrated evidence of acute pulmonary vein reconnection in 45%, 30% and 35.6% of patients (*p* = 0.134). The most common site of residual conduction in case of non-first pass isolation was at the left pulmonary vein carina.

### 3.5 Safety

None of the patients in this study died. Three PAEs were observed in the vHPSD group. One patient experienced an intraprocedural haemorrhagic pericardial effusion with cardiac tamponade, which was immediately treated with pericardiocentesis and initiation of the major haemorrhage protocol, including transfusion of packed red cells and prothrombin complex concentrate. Haemodynamic stability was promptly attained and the patient was transferred to the coronary care unit (CCU) and made a good clinical recovery with no subsequent re-hospitalization noted. One patient experienced a transient episode of junctional rhythm with sinus bradycardia. An exercise tolerance test conducted 48 hours later did not reveal any persistent atrioventricular (AV) block requiring pacemaker insertion. The third event was a 2:1 atrioventricular block that occurred following the ablation of a slow pathway subsequent to PVI for treatment of incessant atrioventricular nodal reentrant tachycardia (AVRNT) and paroxysmal atrial fibrillation (PAF). The PVI component of the procedure was performed with vHPSD while the slow pathway ablation was conducted by conventional ablation setting. The patient developed 2:1 AV block following slow pathway ablation for AVNRT. A temporary pacing wire was inserted followed by a dual chamber permanent pacemaker a few days later. There was no re-admission for procedure-related adverse events.

One PAE was noted in the HPSD group. A cardiac tamponade occurred which required immediate pericardial drain insertion. The patient made a full recovery following a period of monitoring in a high-dependency unit setting.

Four PAEs were observed in the STSF group; one cardiac tamponade, one pericardial effusion not requiring drainage, one access site haematoma and one suspected left anterior circulation stroke. The cardiac tamponade occurred following a transoesophageal echocardiogram and PVI under general anaesthesia, a post-procedural transthoracic echocardiogram (TTE) identified a severe pericardial effusion that required a pericardial drain, following which the patient had an uneventful recovery. The suspected cerebrovascular accident (CVA) occurred following PVI as the patient experienced left-sided weakness and was subsequently transferred to the stroke unit. Following a period of observation, the patient made a full recovery. The access site haematoma resolved with manual compression. One small pericardial effusion was noted on routine post-procedure TTE. A period of monitoring followed by a repeat TTE the next day revealed no evidence of progression of the pericardial effusion or haemodynamic compromise and hence did not require treatment.

## 4. Discussion

We have presented our real-world data in the use of the new vHPSD RF ablation technology in patients undergoing AF ablation focusing on intraprocedural data on PVI. We have found that (1) vHPSD ablation is effective in achieving PVI with a comparable safety profile to ablation using standard power mode; (2) The number of lesions deployed in vHPSD for PVI was higher but the total ablation time shorter which was translated into shorter duration required to achieve PVI compared with standard power mode; (3) There was a reduction in requirement of additional sedative/analgesics during the procedure with vHPSD.

RF ablation has undergone technological development from non-irrigated to irrigated. Irrigation was originally developed to provide more power for penetration during CTI ablation however it also addressed the issue of clot formation during ablation that was noted with prior catheters. In spite of the advantages of open-irrigated catheters, there were impediments due to the change in the geometry of lesions generated compared to those formed via non-irrigated catheters. A reduced area of endocardial surface is exposed to threshold temperatures required for tissue destruction and with that brings forward the concern of relative endocardial sparing [11]. Contiguity of lesions were compromised with lesions starting deeper. Furthermore, enhanced depth of lesions is not always favourable especially when considering thin-walled tissue that lay in precariously close proximity to structures such as the oesophagus, increasing the risk for collateral damage.

Contact force (CF) - sensing catheters were developed to guide the operator with real-time direct measures of contact as opposed to indirect means such as fluoroscopic guidance and tactile feedback [12]. During conventional RF ablation, the application of RF current across the catheter tip results in a shell of resistive heating that catalyses conducting heat to the myocardium. Conductive heating is dependent of duration, current applied and heat generated during the resistive heating. Typically, irreversible myocardial tissue destruction with cellular death ensues at temperatures above 50 degrees Celsius. vHPSD RF ablation modifies the relationship between resistive and conductive heating by applying greater emphasis on the resistive heating phase in order to apply immediate heating to the full thickness of pulmonary vein circumference. As a result, the dependence on conductive heating is limited and collateral tissue damage is restricted.

Leshem and colleagues [5] conducted a study to evaluate the biophysical properties of high-power and short-duration lesions on swine models compared to standard RF ablation. The study employed the QDOT catheter which incorporates additional temperature micro-sensors in close proximity to the catheter tip surface to better ascertain tissue temperatures. This in combination with flow-down titration will allow more resistive heating while limiting the risk of overheating or clot formation. The authors reported 100% contiguity with HPSD ablation while the standard ablation arm observed linear gaps and partial thickness lesions in 25% and 29%, respectively. HPSD generated wider lesions (6.02 ± 0.2 mm vs. 4.43 ± 1.0 mm) at comparable depths (3.58 ± 0.3 mm vs. 3.53 ± 0.6 mm) and the optimal setting determined was 90 W/4 s as it was deemed to exhibit the best compromise between lesion formation and safety. This preclinical model along with early clinic studies [13, 14] have demonstrated favourable efficacy and safety outcomes, however, its efficacy in thicker myocardial regions such as the region around the carina between the upper and lower pulmonary veins, may require further evaluation [15].

The vHPSD treatment group demonstrated shorter time required for PVI and RF energy application in comparison to standard power ablation which is comparable to other study findings on vHPSD technology in literature (Table 4, Ref. [9, 13, 14, 16, 17, 18]). Halbfass *et al*. [14] exhibited the largest patient sample size of 90 patients while total number of applications across the studies ranged from 85 (72–92) to 108. Total procedure time across the studies ranged from 55 (51–62) to 105 minutes while 3 studies reported adverse events in the vHPSD arm with no incidence of cardiac tamponade. Only 1 of the published studies conveyed specific information corresponding to analgesic and sedation requirement [16]. In comparison, our study has utilised a greater sample size with a control arm present. Our patients required a similar number of lesions while exhibiting a substantially lower procedure duration compared to all but one study (Tilz *et al*. [9]). A potential explanation for this difference is the greater sample size of cases and thus exposure for operators to gain experience and conditioning with this power setting. As a result, this acclimatization may facilitate better procedural times and outcomes. The commercial manufacturer’s recommendations were greater overlap of lesions (distance less than 6 mm) in regions where the tissue was expected to be thicker e.g., left atrial appendage (LAA) ridge and anterior wall, which may explain the higher number of lesions used for PVI in comparison to the standard power mode.

**Table 4.** **Studies reporting procedural outcomes of very high-power short duration ablation for atrial fibrillation**.

*Study	Year	Country/region	vHPSD sample size	Control group (n)	Total number of applications		Total procedure time/min		Adverse events (%)	
					vHPSD	Control	vHPSD	Control	vHPSD	Control
QDOT-FAST Trial – Reddy *et al*. [13]	2019	United States	52	N/A	108.3 ± 42.5	N/A	105.2 ± 24.7	N/A	2 (Thromboembolism (1), major vascular access complication (1))	N/A
Tilz *et al*. [9]	2021	Germany	28	Conventional CF-sensing catheter (28)	85 (72–92)	82 (58–110)	55 (51–62)	105 (92–120)	2 (7) – Severe bleeding (1), post procedural pulmonary oedema (1)	1 (4) – Severe bleeding
Halbfass *et al*. [14]	2022	Germany	90	N/A	N/A	N/A	95.5 ± 28.8	N/A	1 (1.11) – Atrioventricular block type II Mobitz – Pacemaker implantation	N/A
Mueller *et al*. [17]	2022	Germany	34	N/A	N/A	N/A	102 ± 32.5	N/A	0	N/A
Bortone *et al*. [18]	2022	France	150	Conventional CF-sensing catheter – QMODE with QDOT catheter (50 W setting)	N/A	N/A	60 (50–70)	65 (60–75)	1 (1.3) – Cardiac tamponade	1 (1.3) – Cardiac tamponade
Chu *et al*. [16]	2023	United Kingdom	59	Conventional CF-sensing catheter and Cryo-ablation	N/A	N/A	118 ± 31	128 ± 40 (Conventional RF), 92 ± 25 (Cryo)	1 (1.7) – Transient global amnesia – managed as a transient ischaemic attack (TIA), 1 (1.7) – Cardiac tamponade – pericardiocentesis	Conventional RF: 1 (1.6) – Cardiac tamponade – pericardiocentesis, 1 (1.6) – Transient dysphagia, 1 (1.6) – Lower respiratory tract infection, Cryo: 1 (1.6) – Cardiac tamponade – pericardiocentesis
Mavilakandy *et al*.**	Current study	United Kingdom	100	Conventional CF-sensing catheter – QMODE with QDOT catheter (30) and Standard RF ablation via STSF catheter (45)	111 (96.7–129.3)	QMODE via QDOT catheter: 93 (80.25–112.5); STSF catheter: 85 (74.5–99.5)	67.7 ± 29.7	QMODE via QDOT catheter: 92.9 ± 25.7; STSF catheter: 93.6 ± 29.1	3 (3) – Cardiac tamponade (1), junctional rhythm with sinus bradycardia (1), atrioventricular block 2:1 – Pacemaker implantation	QMODE via QDOT catheter: 1 (3.33) – Cardiac tamponade STSF catheter: 4 (8.88) – cardiac tamponade (1), pericardial effusion (1), vascular access site complication (1) and suspected cerebrovascular accident (1)

vHPSD, very-high power short duration; n, sample size; N/A, not available/applicable; CF, contact force; RF, radiofrequency; STSF, THERMOCOOL SMARTTOUCH™ SF. * No analgesia/sedation specific information was provided in the first 4 listed. ** Findings from current study – listed for comparison of outcomes with listed studies.

First-pass isolation and acute PV reconnection outcomes were similar to a study conducted by Bortone *et al*. [18] in which the authors compared lesion formation between the 90 W and 50 W settings. The authors reported a lower first-pass PVI rate (49% versus 81%) and greater acute PV reconnection rate (21% versus 5%) [18]. The lower first-pass PVI rate has been previously alluded to with lesion geometry and catheter stability considered as potential explanations. Bortone *et al*. [18] postulated that the higher incidence of conduction gaps was an effect of smaller lesion size (depth and diameter) in comparison to the other power setting especially given the optimal CF and interlesion distance recorded.

Our study observed 3 complications while the other studies ranged from 0–2 complications which could be explained by the greater sample size but the complications are in line with what is expected of an AF ablation and do not appear to be associated with the ablation modality as such. Lastly, our study presents additional data corresponding to analgesic and sedation requirements which has not previously been reported. Reduction in analgesia and sedation requirement compared to the control group potentially suggested greater patient tolerability and comfort during the procedure. These findings could be explained by the reduced duration of RF application for each lesion and the total procedure.

### Study Limitations

The authors acknowledge several limitations in this service evaluation study. Firstly, due to the novelty and recent introduction of this technology, the number of available cases to contribute to the sample size was limited. Patients were prospectively recruited consecutively. The two-year inclusion period was during the COVID-19 pandemic where there was significant disruption to facilitation of PVI procedures. Additionally, other PVI procedures were performed by other ablation technologies such as Cryoablation and some RF ablation procedures were performed with other mapping system. The decision in determining between available ablation options were typically operator dependent. With respect to the control population, the authors felt the sample size was sufficient for comparison. Secondly, due to the service evaluation essence of this study, a formal randomisation process for patient selection and allocation was not employed. Furthermore, with regards to the ablation procedure, multiple different operators contributed to the sample size and thus introduced an inevitable degree of heterogeneity with regard to the technicalities of the intervention. Three operators with significant experience in catheter ablation ranging from 5 to 20 years, participated in the study. Most importantly, as an early service evaluation into real-world feasibility, this study does not provide data for longitudinal outcomes.

Thus, it would be prudent to eventually pursue a study design with a well-defined protocol and appropriate sample size determined from statistical power calculations. More importantly, a longitudinal follow-up period of at least 12 months would be beneficial to ascertaining longer term outcomes corresponding to overall effectiveness in reducing sinus remission.

## 5. Conclusions

This real-world observational study demonstrates vHPSD RFA for PVI in AF demonstrates superior procedural metrics of shorter procedural time, reduced analgesia and sedation requirement along with a comparable safety profile to HPSD RFA and STSF RFA. The widespread adoption of vHPSD can in turn lead to efficiency gains within electrophysiology (EP) services and better patient experiences during AF PVI RFA procedures.

## Data Availability

The datasets used and/or analysed during the current study are available from the corresponding author on reasonable request.
